# Lipoprotein(a) Levels and Embolic Stroke of Undetermined Source in Patients with Patent Foramen Ovale

**DOI:** 10.3390/jcm15166197

**Published:** 2026-08-10

**Authors:** Johannes Bernhard, Johanna Ebner, Lukas Galli, Bea Goessinger, Harald Gabriel, Lore Schrutka, Patrick Haider, Christian Hengstenberg, Konstantin A. Krychtiuk, Stefan Greisenegger, Walter S. Speidl

**Affiliations:** 1 Department of Internal Medicine II, Division of Cardiology, Medical University of Vienna, 1090 Vienna, Austria; johannes.bernhard@meduniwien.ac.at (J.B.);; 2 Department of Neurology, Medical University of Vienna, 1090 Vienna, Austria; 3 Ludwig Boltzmann Institute for Cardiovascular Research, 1090 Vienna, Austria

**Keywords:** embolic stroke, lipoprotein(a), patent foramen ovale

## Abstract

**Background**: Elevated levels of lipoprotein(a) [Lp(a)] are an independent risk factor for the development of atherosclerotic cardiovascular disease (ASCVD). However, there is only limited data investigating the role of elevated Lp(a) levels in patients with patent foramen ovale (PFO)-associated embolic stroke of undetermined source (ESUS). **Methods**: We included 266 patients with PFO-associated ESUS who underwent percutaneous PFO closure and had Lp(a) measurements available. These patients were matched according to age, sex and previously documented statin treatment to 947 hospital controls without stroke. **Results**: Median age was 51 years (IQR 43–59) and 40% were female. Median Lp(a) levels were higher in cases (24 IQR 12–88 nmol/L) than in controls (21 IQR 7–75 nmol/L; *p* = 0.003). This association appeared more pronounced in men than in women. In males, patients with Lp(a) in the second tertile had an odds ratio (OR) of 1.64 (95% CI, 1.03–2.62) and patients with Lp(a) in the third tertile had an odds ratio of 2.18 (95% CI 1.38–3.44) independent of age, statin use, HbA1c and eGFR. In contrast, in females the second (OR 2.08 [95% CI 1.22–3.54]) but not the third tertile (OR 0.88 [95% CI 0.49–1.59]) of Lp(a) was associated with case status. **Conclusions**: In this retrospective matched case–control study, higher Lp(a) concentrations were associated with case status in a selected cohort of patients with PFO-associated ESUS, particularly in men. These findings should be considered hypothesis-generating and require confirmation in prospective studies with more representative comparison cohorts.

## 1. Introduction

Cryptogenic stroke is defined as an ischemic stroke for which conventional etiologies, including large-artery atherosclerosis, small-vessel occlusion (lacunar stroke), hypercoagulable conditions, arterial dissection and established cardioembolic sources have been definitively excluded. Of note, the clinical concept of “embolic stroke of undetermined source” (ESUS) is currently utilized more frequently, as it represents a more specific and defined subset of the broader categorization of cryptogenic stroke [1]. 

Subsequent diagnostic evaluation typically involves the use of transesophageal echocardiography (TOE) to detect any right-to-left shunts through a patent foramen ovale (PFO). Where such shunt is identified, current clinical practice guidelines recommend percutaneous PFO closure to mitigate the risk of recurrent stroke [2].

Of interest, a recent study has shown that patients with ESUS exhibit a higher prevalence of high-risk, non-stenotic intracranial atherosclerotic plaque—currently undetected by routine clinical practice—than those with small-vessel disease (SVD) [3]. Previously published data could also demonstrate a correlation between ipsilateral non-stenotic carotid plaque and ESUS, further highlighting the potential role of atherosclerotic vascular disease in the development of cryptogenic stroke [4].

Beyond structural plaque characteristics, dyslipidemia may contribute to ESUS through the development of subclinical or non-stenotic atherosclerotic disease [3]. Conventional lipid parameters, including low-density lipoprotein (LDL)-cholesterol and high-density lipoprotein (HDL)-cholesterol, are established markers of atherosclerotic vascular risk, although their specific contribution to ESUS remains incompletely defined. This is particularly relevant because ESUS represents a heterogeneous clinical construct in which covert atherosclerosis, atrial cardiopathy, paradoxical embolism, and other embolic mechanisms may overlap [5].

Lipoprotein(a) [Lp(a)] is composed of a low-density lipoprotein (LDL) particle which is covalently linked via the apolipoprotein B-100 (ApoB) to apolipoprotein (a) [apo(a)]. Plasma levels of Lp(a) are primarily governed by the LPA gene, which accounts for 90% of its variability and leads to relatively stable concentrations throughout an individual’s lifetime [6]. Although the physiological function of Lp(a) remains elusive, current evidence suggests that it has a pathogenic role in the development of atherosclerosis and thrombosis [7]. In contemporary cardiovascular prevention, elevated Lp(a) is primarily considered a genetically determined risk modifier and marker of increased atherosclerotic cardiovascular risk, with current clinical use mainly directed toward risk stratification and optimization of global cardiovascular risk management [7]. Apart from investigational products currently in phase three clinical trials and PCSK9-inhibitors that offer modest Lp(a)-lowering, no approved pharmaceutical treatment options are available for affected individuals to specifically and substantially lower Lp(a) in routine clinical practice. Lp(a) apheresis, an invasive extracorporeal blood-filtering procedure, remains the last resort of treatment for progressive atherosclerotic disease [8]. Due to the significant cost and logistics of such treatment, country-specific cut-off values exist, therefore enabling this therapeutic approach to only very high-risk individuals.

To date, data investigating the relationship of plasma Lp(a) concentrations with cryptogenic stroke or ESUS remain limited. One prospective study investigating the relationship of Lp(a) and unexplained stroke in young- and middle-aged adults with a focus on racial-ethnic subgroups could demonstrate an increased odds ratio (OR) in patients with higher Lp(a) levels. However, no effect modification by sex could be shown [9]. In addition, a previous case report described recurrent embolic strokes of undetermined source in a patient with extreme Lp(a) concentrations, suggesting a potential link between markedly elevated Lp(a), non-stenotic atherosclerotic disease, and embolic stroke mechanisms [10]. However, systematic data evaluating Lp(a) concentrations in patients with PFO-associated ESUS undergoing catheter-based PFO closure remain scarce, and sex-stratified analyses in this specific clinical setting are lacking.

Against this background, we aimed to investigate the association between serum levels of Lp(a) and case status in patients with PFO-associated ESUS undergoing catheter-based PFO closure compared with matched hospital controls, with a particular focus on sex-stratified associations.

## 2. Methods

### 2.1. Study Population

Between April 2010 and February 2024, 1424 individual patients underwent catheter-based PFO closure at the Department of Cardiology, Medical University of Vienna. Of these, 692 individuals had a centrally documented Lp(a) value available in nmol/L and were included in the present analysis. An additional 121 patients had Lp(a) values in mg/dL and were not included to avoid assumptions related to unit conversion.

The clinical work-up and detailed procedural techniques of PFO closure have been previously described [11].

Each patient was separately screened by two different neurologists (J.E. and S.G.), who were blinded to Lp(a) values, to confirm fulfillment of ESUS criteria [5]. Any discrepancies were resolved through mutual consensus. Patients with determined cause of stroke, transient ischemic attack (TIA) or other indications for closure of PFO were then excluded (n = 426).

**Figure 1:**
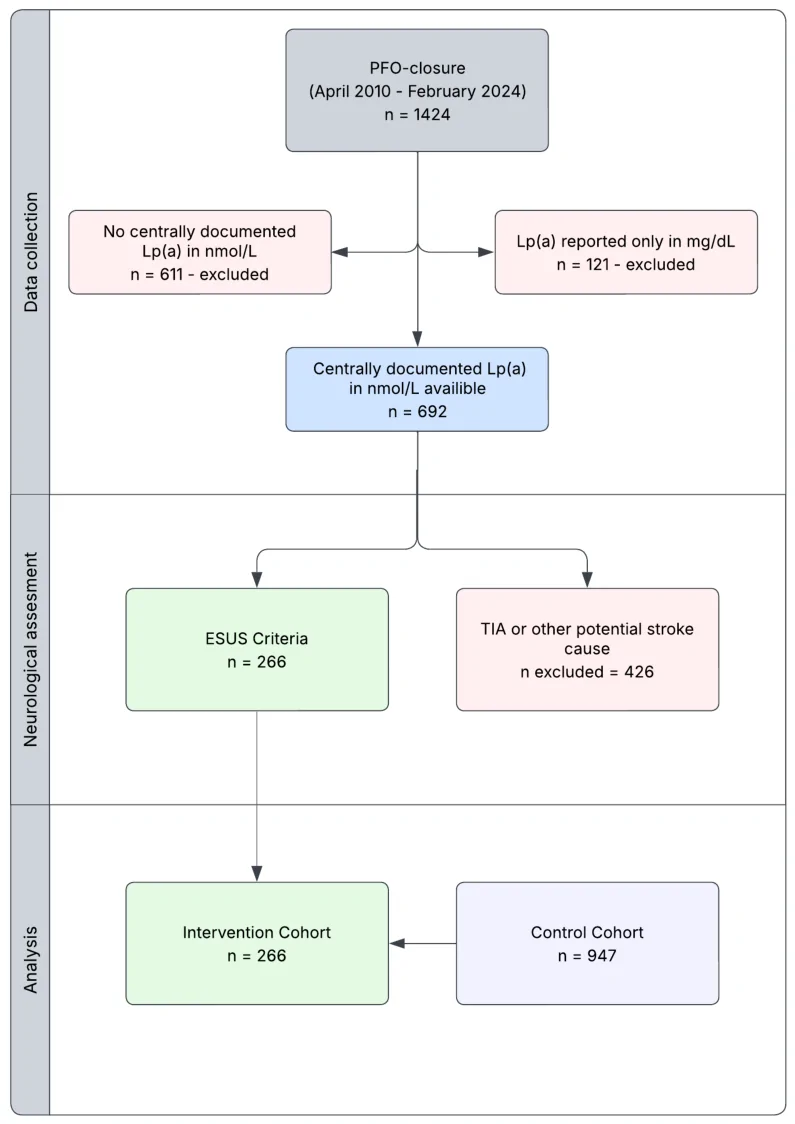
Flowchart of patient selection. PFO, patent foramen ovale; Lp(a), lipoprotein(a); ESUS, embolic stroke of undetermined source; TIA, transient ischemic attack.

In total, 266 patients with PFO-associated ESUS were then matched to 947 hospital controls according to age, sex, and previously documented treatment with statins. Variable-ratio matching was performed with up to four controls per case. If fewer than four eligible controls were available for a given case, all available controls were included. Matching was performed without replacement. Because exact age matching was performed, no age caliper was applied. Accordingly, the comparator group consisted of hospital controls without stroke and without systematic assessment for PFO and was selected to provide a matched reference population rather than a mechanistically equivalent comparison group. The hospital control cohort was automatically generated via the hospital- wide database of the General Hospital of Vienna (N = 155,589 potential control patients with Lp(a) measurement in nmol/L). Lp(a) measurements in the control cohort were obtained during routine clinical care and cardiovascular risk assessment. Figure 1 illustrates the patient selection flowchart.

The Risk of Paradoxical Embolism (RoPE) score, the PFO-Associated Stroke Causal Likelihood (PASCAL) classification system and PFO morphology were retrospectively gathered for patients in the intervention group via the hospital database [12,13]. A patent foramen ovale (PFO) was deemed large when either ≥20 microbubbles were observed or a left-to-right shunt was unmistakably seen in TOE without a Valsalva maneuver. Septal abnormalities were recorded, with an atrial septal aneurysm (ASA) defined as a septal excursion of ≥10 mm from the midline.

### 2.2. Blood Sampling and Laboratory Analysis

In patients undergoing PFO occlusion which were included in this study, blood was drawn directly after admission to the medical ward or in the morning prior to PFO closure when admission to the medical ward was after 2 pm. Because of the retrospective design, blood sampling was not standardized to a fixed interval after the index stroke. Standard laboratory measurements were analyzed in the ISO certified central laboratory of the General Hospital of Vienna. Total cholesterol (TC), HDL-C, and triglycerides were measured by enzymatic methods and stated in mg/dL. LDL-C was calculated using the Friedewald formula and stated in mg/dL. Estimated glomerular filtration rate (eGFR) was calculated by the Modification of Diet in Renal Disease (MDRD) formula. Lp(a) measurement was performed using the Tina-quant Lipoprotein(a) Gen.2 assay (Roche Diagnostics International AG, Rotkreuz, Switzerland). This assay is not affected or minimally affected by apo(a) size variation and is calibrated in nmol/L.

### 2.3. Statistical Analysis

Baseline categorical variables were summarized as counts and percentages and compared using the χ^2^ test or Fisher’s exact test, as appropriate. Continuous variables were expressed as median (interquartile range) and compared using the Mann–Whitney U test. Correlations were assessed using Spearman’s rank correlation coefficient.

Multivariable logistic regression models were used to evaluate the association between Lp(a) and case status. Models were adjusted for age, sex, prior statin use, HbA1c, and eGFR. Odds ratios (ORs) for case status were calculated across Lp(a) tertiles. Interactions were tested by including interaction terms in the logistic models, with *p* < 0.10 considered statistically significant. To explore potential sex-specific associations, separate models were fitted for men and women.

Natural cubic spline logistic regression models with three degrees of freedom were used to assess potential non-linear associations between Lp(a) and case status. Internal knots were located at 13 and 58 nmol/L, with boundary knots at 7 and 719 nmol/L. The reference value for odds ratios was set at 7 nmol/L. All available Lp(a) values were included in the models; figures were restricted to <7–400 nmol/L for visualization.

Models were adjusted for age, sex, prior statin use, HbA1c, and eGFR. Sex-stratified models excluded sex as a covariate. Sensitivity analyses were performed as complete-case analyses according to covariate availability. Covariate balance was assessed using standardized mean differences. Conditional logistic regression stratified by matched set was used to account for the matched design. The multivariable logistic regression model was considered the primary regression analysis. Conditional logistic regression stratified by matched set was used as a matched-design sensitivity analysis and was restricted to informative matched sets with complete covariate data. Additional models evaluated clinically interpretable Lp(a) thresholds and sex-by-Lp(a) interactions. Exploratory analyses restricted to patients with PFO-associated ESUS assessed associations between Lp(a) and RoPE score, PASCAL classification, and available PFO morphology variables.

Two-sided *p* values < 0.05 were considered statistically significant. Threshold, spline and interaction analyses were considered exploratory. All analyses were performed using SPSS version 22.0 (IBM Corporation, Armonk, NY, USA) and R version 4.2.3 (R Foundation for Statistical Computing, Vienna, Austria).

### 2.4. Artificial Intelligence (AI)-Assisted Technology

During the preparation of this work, the authors used ChatGPT (GPT-5.5 Thinking; OpenAI, San Francisco, CA, USA) for language editing, including improvements in clarity, grammar, and spelling. The tool was not used for data generation, image generation, or scientific interpretation. The authors reviewed and edited all AI-assisted suggestions and take full responsibility for the content of the published article.

## 3. Results

### 3.1. Baseline Characteristics

We included 266 patients with PFO-associated ESUS who underwent closure of a patent foramen ovale and had Lp(a) measurement available. These patients were matched according to age, sex and previously documented statin use to 947 hospital controls. Covariate balance is shown in Supplementary Table S1.

Median age was 51 years (IQR 43–59) and 40% were female. At the time of Lp(a) measurement, 55.2% of patients were on statin treatment. Lp(a) values ranged from <7.0 to 511 nmol/L in the ESUS cohort and from <7.0 to 747 nmol/L in hospital controls (Table 1).

Patients with PFO-associated ESUS had a mean RoPE Score of 6.2, with 44.0% of patients scoring at or above 7 points. After adding the specific criteria of the PASCAL classification system, the PFOs of 25.9% of patients were classified as probable, 57.1% as possible and 15.4% as an unlikely cause of the index stroke.

### 3.2. Plasma Levels of Lipoprotein(a) in Patients with PFO-Associated Embolic Stroke of Undetermined Source

**Figure 2:**
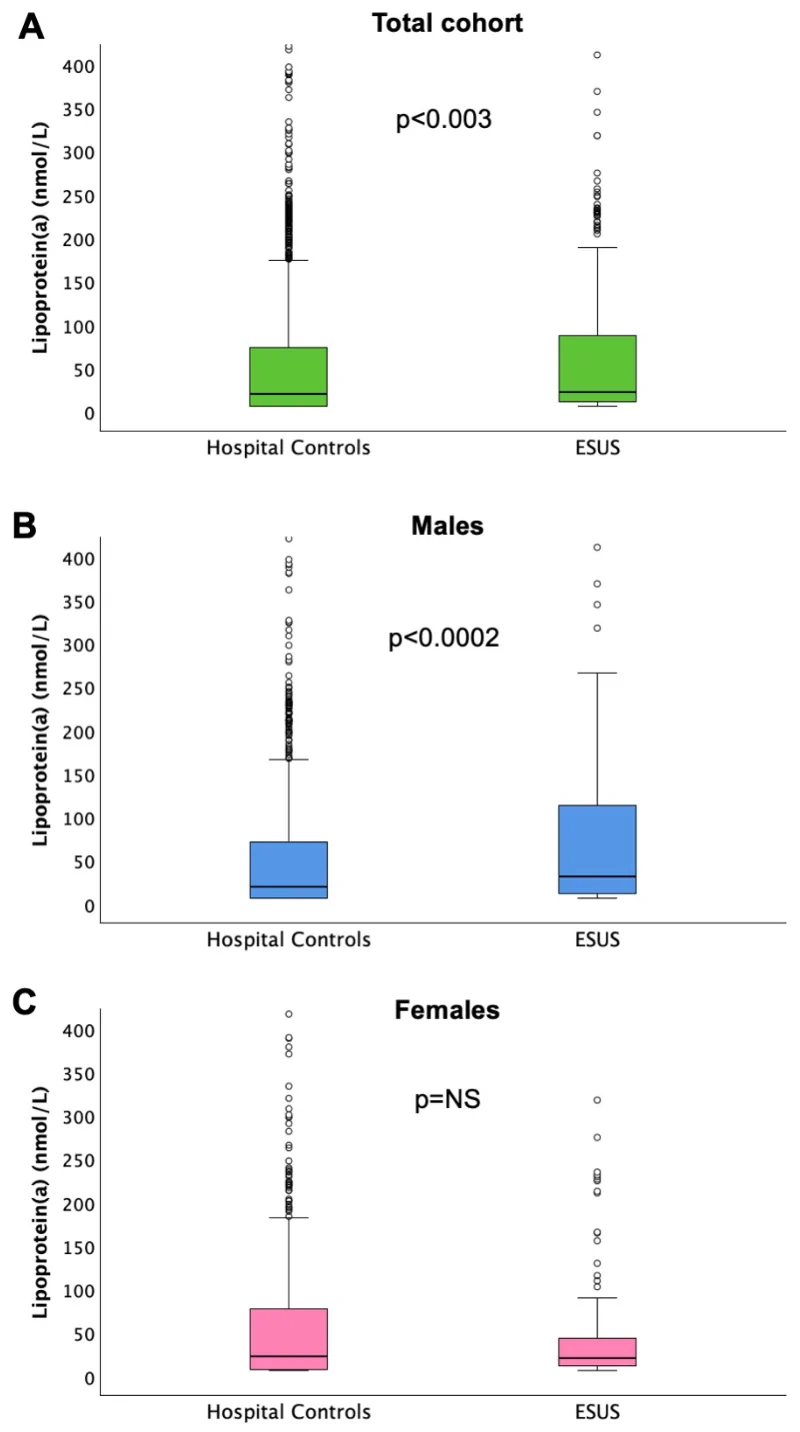
Distribution of Lipoprotein(a) by case–control status for the total cohort (**A**), male patients (**B**) and female patients (**C**). Only Lp(a) values from <7 to 400 nmol/L are shown. ESUS embolic stroke of undetermined source.

In patients with PFO-associated ESUS, median Lp(a) levels were higher (24 (IQR 12–88) nmol/L) than hospital controls without stroke (21 (IQR 7–75) nmol/L; *p* = 0.003). Interestingly, this difference between patients and controls in Lp(a) plasma levels was more pronounced in male patients (ESUS: 32 (IQR 12–114) nmol/L vs. controls: 20 (IQR 7–71) nmol/L; *p* < 0.001) but could not be detected in female patients (ESUS: 21 (IQR 12–44) nmol/L vs. controls: 23 (IQR 8–78 nmol/L); *p* = 0.97; Figure 2).

### 3.3. Sex-Specific Association of Lipoprotein(a) with PFO-Associated Embolic Stroke of Undetermined Source

**Figure 3:**
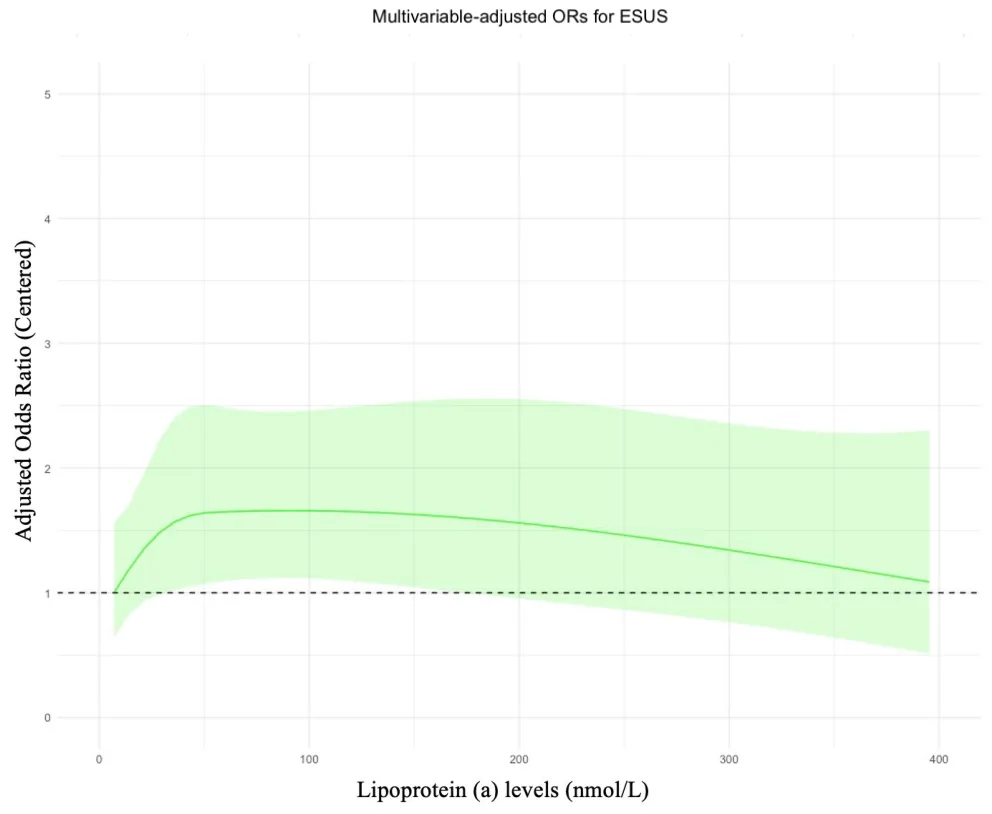
Multivariable-adjusted odds ratios (OR) for case status according to lipoprotein(a) levels on a continuous scale. Multivariable model is adjusted for age, sex, statin use, hemoglobin A1c and glomerular filtration rate. Lp(a) levels are shown in nmol/L; the displayed *x*-axis range was restricted to <7–400 nmol/L for visualization. ESUS embolic stroke of undetermined source, OR odds ratio.

**Figure 4:**
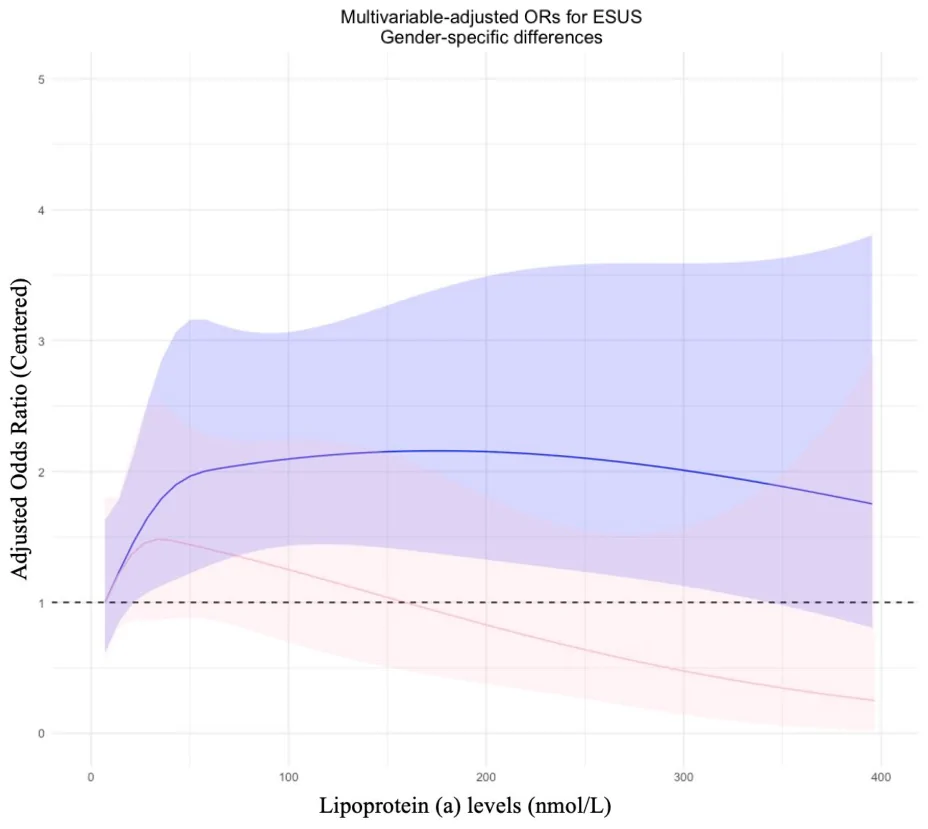
Multivariable-adjusted odds ratios (OR) for case status according to sex and lipoprotein(a) levels on a continuous scale. Males are plotted in blue, females in pink. Multivariable models are adjusted for age, statin use, hemoglobin A1c and glomerular filtration rate. Lp(a) levels are shown in nmol/L; the displayed *x*-axis range was restricted to <7–400 nmol/L for visualization. ESUS embolic stroke of undetermined source, OR odds ratio.

In the total cohort, Lp(a) plasma levels in the second and third tertile (T1 ≤ 11 nmol/L, T2 12–48 nmol/L, T3 ≥ 49 nmol/L) were associated with higher odds of case status (Figure 3). Patients in the second tertile of Lp(a) had an odds ratio (OR) of 1.90 (95% CI 1.33–2.69; *p* < 0.001) and in the third tertile of 1.54 (95% CI 1.08–2.21; *p* = 0.019) after adjustment for age, sex, statin use, HbA1c and eGFR. Continuous associations across the Lp(a) range are shown in Figure 3, and sex-stratified continuous associations are shown in Figure 4.

In males, patients with Lp(a) in the second tertile had an adjusted OR of 1.64 (95% CI, 1.03–2.62; *p* = 0.037) and patients with Lp(a) in the third tertile had an adjusted OR of 2.18 (95% CI 1.38–3.44; *p* < 0.001) for case status. Female patients in the second tertile of Lp(a) had an adjusted OR of 2.08 (95% CI 1.22–3.54; *p* < 0.01). In female patients, Lp(a) levels in the third tertile were not associated with case status (OR 0.88, 95% CI 0.49–1.59; *p* = 0.67).

In complete-case sensitivity analyses, the association between log2-transformed Lp(a) and case status remained directionally positive but did not reach statistical significance in conventional logistic regression (700 individuals from 255 matched sets; OR 1.08, 95% CI 0.981–1.19; *p* = 0.112). Conditional logistic regression stratified by matched set showed similar directionally positive but non-significant associations for log2-transformed Lp(a) (487 individuals from 154 informative matched sets; OR 1.09, 95% CI 0.981–1.22; *p* = 0.105), Lp(a) ≥ 75 nmol/L (OR 1.35, 95% CI 0.875–2.09; *p* = 0.174), and Lp(a) ≥ 125 nmol/L (OR 1.07, 95% CI 0.667–1.72; *p* = 0.780; Supplementary Table S2).

Clinically relevant Lp(a) thresholds were not significantly associated with case status in the overall cohort, including Lp(a) ≥ 75 nmol/L, ≥125 nmol/L, and the 95th percentile threshold of 255.8 nmol/L. In sex-stratified analyses, Lp(a) ≥ 75 nmol/L was associated with higher odds of case status in men (OR 1.70, 95% CI 1.16–2.48; *p* = 0.006), but not in women.

Formal interaction testing supported sex-related heterogeneity for log2-transformed Lp(a) (*p* for interaction = 0.043) and for threshold-based analyses using Lp(a) ≥ 75 nmol/L (*p* = 0.0017) and ≥125 nmol/L (*p* = 0.012); whereas, the interaction for Lp(a) tertiles was not statistically significant (*p* = 0.105).

To analyze possible sex-specific differences in PFO-related clinical characteristics, we compared RoPE score and PASCAL classification in male and female patients. Mean RoPE Score was 6.2 ± 1.7 in males and 6.3 ± 1.6 in females (*p* = 0.62). PASCAL classification did not differ between males and females (*p* = 0.98). In addition, PFO tunnel length (*p* = 0.68) and separation (*p* = 0.93) did not show sex-specific differences. Similarly, the presence of large shunts was 61.0% and 57.6% in males and females (*p* = 0.41) and atrial septal aneurysms were found in 16.4% of males and 19.2% of females (*p* = 0.39).

Lp(a) levels also did not differ significantly according to RoPE score category, PASCAL classification, or PFO morphology. Median Lp(a) was 29 nmol/L (IQR 14–103) in patients with RoPE score < 7 and 21 nmol/L (IQR 11–75) in patients with RoPE score ≥ 7 (*p* = 0.073). Lp(a) levels did not differ by PASCAL classification (*p* = 0.185), large-shunt status (*p* = 0.926), atrial septal aneurysm (*p* = 0.890), or hypermobile septum (*p* = 0.845). No meaningful correlation was observed between Lp(a) and PFO length (rho = −0.043; *p* = 0.516) or PFO separation (rho = 0.011; *p* = 0.864).

### 3.4. Sex-Specific Association of Lipoprotein(a) with Age

**Figure 5:**
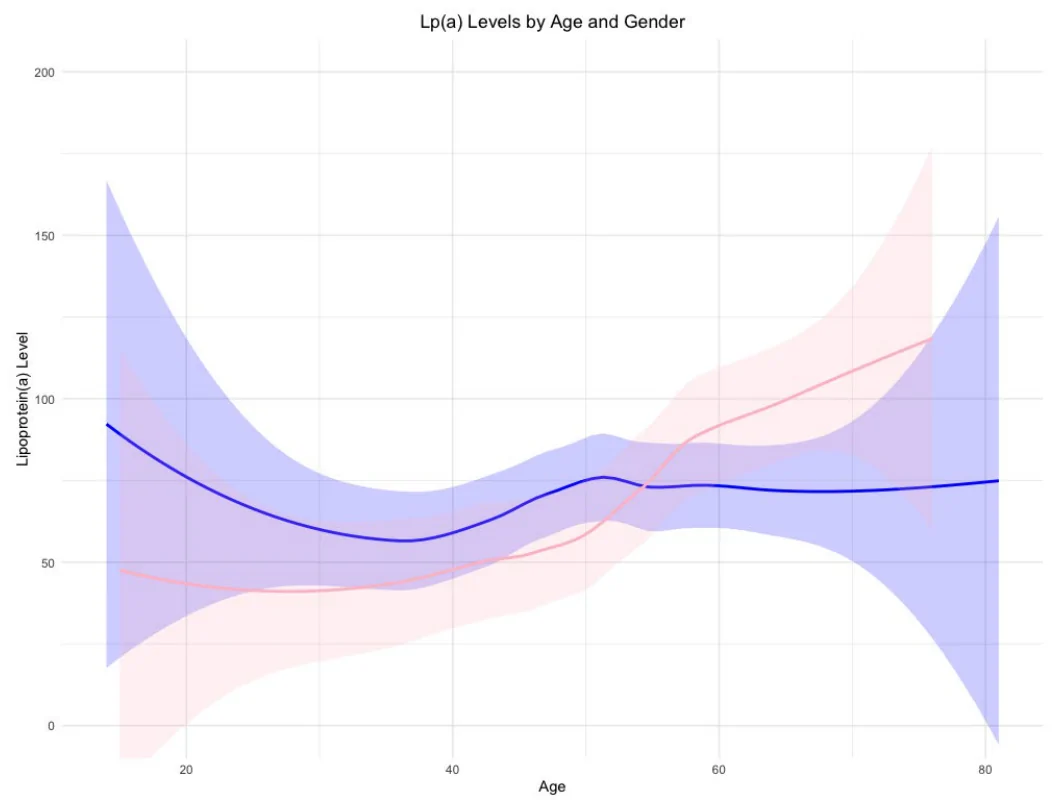
Smoothed trends of lipoprotein(a) levels by age and sex. Males are shown in blue and females in pink. The plot displays continuous associations between age (*x*-axis) and lipoprotein(a) levels (*y*-axis) with 95% confidence intervals around the smoothing lines. Lp(a), Lipoprotein (a).

The sex-specific association of Lp(a) with age is shown in Figure 5. In contrast to males (r = 0.05; *p* = 0.21), Lp(a) plasma levels in females increased with age (r = 0.13; *p* = 0.005). This modest association of Lp(a) plasma levels with age was found only in female controls (r = 0.15; *p* = 0.004) but not in female patients after stroke (r = 0.05; *p* = 0.53). Women aged 50 years or older had higher Lp(a) levels (24 IQR 10 to 86 nmol/L) as compared to women < 50 years of age (21 IQR 8 to 54 nmol/L; *p* < 0.05). There was no significant age difference between the included female and male individuals (*p* = 0.62).

## 4. Discussion

In this retrospective matched case–control study, median Lp(a) concentrations were higher in patients with PFO-associated ESUS who underwent PFO closure than in an age-, sex-, and statin-use-matched cohort of hospital patients. Higher Lp(a) levels were associated with higher odds of case status after adjusting for age, sex, HbA1c, statin use and eGFR. When stratified for sex, the association was more pronounced in male individuals. These findings support an association between Lp(a) and this selected PFO-associated ESUS phenotype, but do not establish Lp(a) as a causal risk factor. Accordingly, the present analysis cannot distinguish whether elevated Lp(a) reflects PFO-related stroke mechanisms, broader ESUS susceptibility, or general vascular risk.

Although median Lp(a) concentrations were statistically higher in patients with PFO-associated ESUS than in matched hospital controls, the absolute difference was modest and there was substantial overlap between groups. The present findings should therefore not be interpreted as indicating that Lp(a) alone can discriminate patients with PFO-associated ESUS from hospital controls at the individual level. In clinically interpretable threshold analyses, Lp(a) ≥ 75 nmol/L, ≥125 nmol/L, and the 95th percentile threshold were not significantly associated with case status in the overall cohort. However, sex-stratified analyses showed higher odds of case status among men with Lp(a) ≥ 75 nmol/L; whereas, no positive association was observed in women. These findings support a cautious interpretation of the observed association as a modest, sex-dependent population-level signal rather than a robust threshold-dependent relationship. The findings of the tertile-based analysis should also be interpreted cautiously and in the context of the performed sensitivity analysis, in which the associations were directionally similar but did not reach statistical significance.

The classification of the index event as ESUS, even though all patients had a diagnosed PFO, was made because under the established ESUS construct a PFO is considered a potential conduit for paradoxical embolism rather than a major-risk cardioembolic source. Accordingly, the PASCAL and RoPE scores estimate the likelihood that the PFO is pathogenic; they do not establish definitive causality. We therefore interpret the present cohort as a selected population with PFO-associated ESUS undergoing closure, rather than as an unselected ESUS population.

In exploratory analyses restricted to the PFO-associated ESUS cohort, Lp(a) levels were not clearly associated with RoPE score, PASCAL classification, or PFO morphology. The absence of associations between Lp(a) and these classification markers argues against a direct relationship between these, although these analyses were exploratory and limited by sample size.

The rationale for exploring the association between Lp(a) and PFO-associated ESUS is based on the potential biological effects of Lp(a) on thrombosis and atherosclerosis. Lp(a) is structurally homologous to plasminogen and has been linked to impaired fibrinolysis and other prothrombotic properties [14]. Additionally, Lp(a) may contribute to endothelial dysfunction and the acceleration of atherosclerosis [7], including the development of non-stenotic atherosclerotic plaque that can serve as an embolic source. However, the biological relevance of Lp(a) in PFO-associated stroke remains uncertain, and the present data do not allow mechanistic inference.

Previous clinical investigations into the relationship between Lp(a) plasma levels and the risk of ischemic stroke have reported conflicting findings. In the prospective EPIC-Norfolk population study, elevated Lp(a) levels were associated with peripheral artery disease (PAD) and coronary artery disease (CAD) events, but not with ischemic stroke [15]. Similarly, data from nearly 15,000 white males from the Physicians’ Health Study also failed to demonstrate any effect of elevated Lp(a) levels on future thromboembolic stroke or stroke of any cause [16]. Interestingly, in the Northern Manhattan Stroke Study, plasma Lp(a) levels ≥ 30 mg/dL were associated with ischemic stroke, even after correcting for known cardiovascular risk factors. However, in a stratified analysis, a significant difference was only observed in males and in black individuals [17]. Partially divergent data were reported in the ARIC Study with elevated Lp(a) levels of ≥30 mg/dL only associated with significantly higher risk of ischemic stroke in black individuals and white females [18]. Furthermore, a comprehensive meta-analysis by Nave et al. (2015) identified elevated Lp(a) as an independent risk factor for ischemic stroke, with a particularly pronounced effect observed in younger individuals [19].

The present study is, to our knowledge, the first to evaluate sex-specific associations of Lp(a) in patients with PFO-associated ESUS undergoing catheter-based PFO closure. Therefore, comparing the presented data to previously published data is challenging, as the potential underlying stroke mechanisms in prior studies may differ substantially from those in the present cohort. In our study, several potential causes of ischemic stroke including major cardioembolic sources, significant large artery atherosclerosis (intra- and extracranial stenosis ≥ 50% in arteries supplying the area of infarct or arterial dissection) and other specific stroke causes were excluded by two experienced neurologists. Notably, all stroke patients in our cohort had a PFO as diagnosed by TOE. As previously mentioned, potential mechanisms of stroke in this cohort may include paradoxical embolism through a PFO as well as non-cardioembolic embolic sources such as non-stenotic atherosclerotic plaque.

Although no specific data on the association of Lp(a) and the occurrence of ESUS has been published, evidence exists from one prospective study supporting our findings. In the THICK study, which prospectively included 255 patients with various ethnic backgrounds between the age of 18–64 with unexplained stroke and 390 stroke free controls, an approximately 2-fold greater odds in the highest quartile compared to the lowest quartile of Lp(a) in white individuals was shown. Notably, no significant effect modification by sex or age was observed [9].

Further corroborating the association between elevated Lp(a) and ischemic stroke in younger populations, a meta-analysis by Koh et al. demonstrated that young stroke patients (<65 years) exhibited significantly higher Lp(a) levels compared to age-matched controls, suggesting a consistent association between elevated Lp(a) and early-onset stroke [20]. Additionally, a meta-analysis by Borzillo et al. reported that elevated Lp(a) levels in youth and childhood are associated with a more than two-fold increased risk of arterial stroke, underscoring the importance of Lp(a) as a risk factor in younger demographics [21].

Given the postulated pathophysiological effects of Lp(a), the underlying cause of the observed effects remains unclear. In addition to elevated plasma levels of Lp(a) being clearly associated with a greater risk of developing atherosclerosis and ensuing cardiovascular disease [22], an association with cerebral large artery atherosclerosis was also demonstrated in the prospective BIOSIGNAL study [23]. As the presence of ipsilateral intracranial atherosclerotic plaque with <50% stenosis has been associated with ESUS [3], the premature development of a high-risk atherosclerotic lesion potentially leading to ESUS could therefore be attributed to elevated levels of Lp(a). Moreover, Nasr et al. identified a significant association between elevated Lp(a) concentrations and carotid atherosclerosis in young adults with ischemic stroke [24]. Recent data from Caruso et al. demonstrated that elevated Lp(a) levels are independently associated with higher Fazekas scores, indicative of greater white matter hyperintensities (WMHs), in young patients with ischemic stroke or transient ischemic attack [25]. In general, these findings suggest that elevated Lp(a) may contribute to cerebral small and large vessel disease, further highlighting its role in cerebrovascular pathology.

The notable similarity between apo(a) and plasminogen has prompted discussions on various antifibrinolytic and consequently pro-coagulatory mechanisms associated with Lp(a) [14]. In theory therefore, elevated levels of Lp(a) would be expected to increase the risk of developing clinical venous thromboembolism (VTE) such as DVT or pulmonary embolism (PE). However, various case–control studies, prospective studies and genome wide association studies (GWAS) have failed to demonstrate a consistent association, with one combined post hoc analysis showing a significant association only in patients with very high levels of serum Lp(a) [26,27,28,29]. Notably, in children with arterial ischemic stroke (AIS), a cohort in which atherosclerosis is rarely present, one small case–control study demonstrated that elevated Lp(a) levels > 50 mg/dL were associated with higher risk of AIS [30], with another showing higher relative risk of recurrent AIS in patients with Lp(a) levels > 300 mg/dL [31]. In contrast, patients included in our study had a median age of 51 years with a minimum age of 21 at the time of PFO occlusion. These pediatric data suggest that prothrombotic mechanisms may warrant consideration in younger stroke populations; however, their relevance to adults with PFO-associated ESUS remains uncertain.

The pronounced difference in sex demonstrated in our patient cohort was particularly interesting. There were no differences in RoPE and PASCAL scores as well in PFO tunnel length, separation, and presence of large shunts or atrial septal aneurysms in sex-specific analyses, suggesting similar PFO morphology and risk for ESUS in both sexes.

As demonstrated in our cohort, increasing age was associated with increasing Lp(a) levels in females but not in the male cohort. This finding is in line with recent literature, which showed higher levels of plasma Lp(a) in females after menopause [32]. In general, it is well established that women, particularly at younger ages, have a lower risk of developing cardiovascular disease. However, the benefit on cardiovascular health fades after women reach menopause, which can be attributed to lower levels of circulating estrogen [33]. Previous studies on Lp(a) and ischemic stroke have reported heterogeneous sex-stratified findings, including associations in selected male subgroups, associations in women, and studies without clear effect modification by sex, suggesting that sex-specific patterns may depend on population characteristics, age distribution, ethnicity, and residual vascular risk [9,17,18]. The sex-stratified findings should nevertheless be interpreted cautiously. Although formal interaction testing supported sex-related heterogeneity for continuous Lp(a) and several threshold-based analyses, the interaction for Lp(a) tertiles was not statistically significant. In addition, the non-monotonic association observed in women, with higher odds in the second but not the third Lp(a) tertile, does not support a simple dose–response relationship. Given the smaller female subgroup size, residual confounding, and potential selection bias in the hospital control cohort, this pattern may reflect limited power or chance rather than a true biological U-shaped relationship. Another possible explanation is selection bias in the female control group, which included a relatively high proportion of individuals on statin therapy and may therefore have been enriched for patients in whom Lp(a) was measured because of suspected or established atherosclerotic disease. This could have attenuated differences between groups, although this interpretation remains speculative.

### Limitations

Several limitations warrant consideration. First, the comparator choice is a central limitation. Cases had PFO-associated ESUS and underwent PFO closure; whereas, controls were hospital patients without stroke and without systematic assessment for PFO. Accordingly, this study cannot determine whether the observed association reflects stroke susceptibility, PFO status, or characteristics of the selected closure population. In addition, age matching alone may not fully account for differences between patients that underwent PFO closure and a general hospital-based control cohort. The retrospective design and reliance on historical records spanning April 2010 to February 2024 may also have introduced variability in data completeness and consistency, although the use of a single Lp(a) assay and blinded retrospective adjudication of stroke etiology by two neurologists support internal validity. Furthermore, selection bias cannot be excluded, as patients without centrally documented Lp(a) values in nmol/L and patients with ESUS but no underlying PFO were not included. In addition, although one-time Lp(a) measurement is recommended for cardiovascular risk assessment, the specific clinical indication for Lp(a) measurement in individual hospital controls could not be systematically reconstructed retrospectively. The control cohort, while matched for age, sex, and statin use, may not fully represent the general population and differed from cases in several baseline characteristics, including dyslipidemia, diabetes, and chronic kidney disease, which may have influenced the observed associations despite adjustment. Residual confounding also remains possible because important cardiovascular risk factors, including blood pressure, smoking status, body mass index, and inflammatory markers, were not available for analysis. In addition, sex-stratified analyses, particularly in women, were based on smaller subgroups and should therefore be interpreted cautiously. Finally, blood sampling was not standardized to a fixed interval after the index stroke, so an influence of acute-phase biology cannot be excluded, and the single-center setting without data on ethnic background may limit generalizability to broader populations.

## 5. Conclusions

In conclusion, in this retrospective matched case–control analysis of 266 patients with PFO-associated ESUS who underwent catheter-based PFO closure versus 947 hospital controls, higher plasma Lp(a) levels were associated with case status, with this association appearing more pronounced in male patients. Given the selected study population, comparator limitations, and potential residual confounding, these findings should be interpreted as exploratory and hypothesis-generating. Prospective studies with mechanistically more appropriate comparator populations are needed to clarify this association.

## Figures and Tables

**Table 1:** Baseline characteristics.

	PFO-Associated ESUS (n = 266)	Hospital Controls (n = 947)	*p* Value
Age, median (IQR)	51 (IQR 43–59)	51 (IQR 43–59)	
Sex			
Male, n (%)	158 (59.4%)	570 (60.2%)	
Female, n (%)	108 (40.6%)	377 (39.8%)	
Lp(a) (nmol/L), median (IQR)	23.5 (IQR 12.0–87.7)	21.0 (IQR 7.0–74.5)	0.003
Statin treatment, n (%)	151 (56.8%)	519 (54.8%)	
HbA1c (%), median (IQR)	5.5 (IQR 5.3–5.5)	5.5 (IQR 5.5–5.7)	<0.001
Total cholesterol (mg/dL), median (IQR)	153.0 (IQR 129.0–183.0)	167.0 (IQR 137.0–206.2)	<0.001
HDL (mg/dL), median (IQR)	54.0 (IQR 45.0–66.0)	48.0 (IQR 38.0–59.0)	<0.001
LDL (mg/dL), median (IQR) †	77.8 (IQR 57.1–106.8)	88.6 (IQR 62.3–119.6)	0.004
Triglycerides (mg/dL), median (IQR)	91.0 (IQR 69.0–128.0)	119 (IQR 84.0–178.0)	<0.001
eGFR (mL/min/1.72 m^2^), median (IQR)	92.4 (IQR 81.8–102.8)	92.4 (IQR 79.1–106.2)	0.392
RoPE Score, mean (SD)	6.2 (1.7)	--	--
RoPE Score ≥ 7, n (%)	117 (44.0%)	--	--
PFO morphology		--	--
Length, mean mm (SD)	12.1 (4.1)		
Separation, mean mm (SD)	2.9 (1.7)		
Large-shunt PFO, n (%)	151 (59.7%)		
Atrial septum aneurysm, n (%)	44 (17.5%)		
Floating septum, n (%)	41 (16.8%)		
PASCAL classification system		--	--
Probable, n (%)	69 (25.9%)		
Possible, n (%)	152 (57.1%)		
Unlikely, n (%)	41 (15.4%)		
Missing, n (%)	4 (1.5%)		

IQR, interquartile range; Lp(a), lipoprotein(a); HDL, high-density lipoprotein; LDL, low-density lipoprotein; eGFR, glomerular filtration rate; RoPE, Risk of Paradoxical Embolism; PFO, patent foramen ovale; PASCAL, PFO-Associated Stroke Causal Likelihood; † LDL value calculated with Friedewald formula.

## Data Availability

Data can be made available to qualified researchers upon reasonable request to the corresponding author and subject to a data use agreement.

## Supplementary Materials

The following supporting information can be downloaded at: https://www.mdpi.com/article/10.3390/jcm15166197/s1, Table S1. Covariate balance in the full matched cohort; Table S2. Complete-case and matched-design sensitivity analyses.
