# Supplementary material for: Lipoprotein(a) Levels and Embolic Stroke of Undetermined Source in Patients with Patent Foramen Ovale
Source: J Clin Med. 2026 Aug 10;15(16):6197. doi: 10.3390/jcm15166197 (PMC13513264; doi:10.3390/jcm15166197)
Supplement: Supplementary file 1 [file jcm-15-06197-s001.zip › jcm-4391081-supplementary.pdf]

Supplementary Data

	Hospital controls	PFO-associated ESUS	SMD
**Age, years, mean**	50.205	50.654	0.038
**Female sex, proportion**	0.398	0.406	0.016
**Prior statin treatment, proportion**	0.548	0.568	0.040
**Lp(a), nmol/L, mean**	69.478	67.113	-0.024
**HbA1c, %, mean**	6.010	5.459	-0.515
**Creatinine, mg/dL, mean**	1.011	0.883	-0.201
**LDL-C, mg/dL, mean**	93.851	84.136	-0.249
**HDL-C, mg/dL, mean**	50.379	56.275	0.368
**Total cholesterol, mg/dL, mean**	173.485	157.761	-0.333
**Triglycerides, mg/dL, mean**	157.089	103.922	-0.406

Supplementary Table S1. Covariate balance in the full matched cohort. Values are shown as means for continuous variables and proportions for binary variables. Standardized mean differences were calculated in the full matched cohort. Absolute SMD <0.10 was considered good balance. Larger imbalances were observed for variables not included in the matching algorithm.

Model	n	Sets	Exposure	OR	95% CI	p value
Complete-case conventional logistic regression	700	255	log2-transformed Lp(a)	1.08	0.981–1.19	0.112
Complete-case conventional logistic regression	700	255	Lp(a) tertile 2 vs 1	1.48	0.931–2.36	0.099
Complete-case conventional logistic regression	700	255	Lp(a) tertile 3 vs 1	1.60	1.02–2.52	0.042
Conditional logistic regression	487	154	log2-transformed Lp(a)	1.09	0.981–1.22	0.105
Conditional logistic regression	487	154	Lp(a) ≥75 nmol/L	1.35	0.875–2.09	0.174
Conditional logistic regression	487	154	Lp(a) ≥125 nmol/L	1.07	0.667–1.72	0.780

Supplementary Table S2. **Complete-case and matched-design sensitivity analyses.** Values are odds ratios with 95% confidence intervals. Complete-case conventional logistic regression models were adjusted for age, sex, prior statin treatment, HbA1c, and renal function. Conditional logistic regression was stratified by matched set and adjusted for HbA1c and renal function; matching variables were not included as covariates. In the full matched cohort, HbA1c data were missing for 502 of 1,213 participants and renal-function data for 128 of 1,213 participants. Conditional logistic regression included 487 individuals from 154 informative matched sets. Lp(a), lipoprotein(a); OR, odds ratio; CI, confidence interval.
